# Microporous Fluorescent Poly(D,L-lactide) Acid–Carbon Nanodot Scaffolds for Bone Tissue Engineering Applications

**DOI:** 10.3390/ma17020449

**Published:** 2024-01-17

**Authors:** Nicolò Mauro, Giovanna Calabrese, Alice Sciortino, Maria G. Rizzo, Fabrizio Messina, Gaetano Giammona, Gennara Cavallaro

**Affiliations:** 1 Department of “Scienze e Tecnologie Biologiche Chimiche e Farmaceutiche” (STEBICEF), Università Degli Studi di Palermo, Via Archirafi 32, 90123 Palermo, Italy; gaetano.giammona@unipa.it (G.G.); gennara.cavallaro@unipa.it (G.C.); 2 Department of Chemical, Biological, Pharmaceutical and Environmental Sciences, University of Messina, Viale Ferdinando Stagno d’Alcontres 31, 98168 Messina, Italy; giovanna.calabrese@unime.it (G.C.); mgrizzo@unime.it (M.G.R.); 3 Department of Chimica e Fisica “E. Segrè”, Università Degli Studi di Palermo, Via Archirafi 36, 90123 Palermo, Italy; alice.sciortino02@unipa.it (A.S.); fabrizio.messina@unipa.it (F.M.)

**Keywords:** PLA, carbon dots, fluorescent biomaterials, bone regeneration, nanocomposites, TIPS

## Abstract

In this study, we introduce novel microporous poly(D,L-lactide) acid–carbon nanodot (PLA-CD) nanocomposite scaffolds tailored for potential applications in image-guided bone regeneration. Our primary objective was to investigate concentration-dependent structural variations and their relevance to cell growth, crucial aspects in bone regeneration. The methods employed included comprehensive characterization techniques such as DSC/TGA, FTIR, rheological, and degradation assessments, providing insights into the scaffolds’ thermoplastic behavior, microstructure, and stability over time. Notably, the PLA-CD scaffolds exhibited distinct self-fluorescence, which persisted after 21 days of incubation, allowing detailed visualization in various multicolor modalities. Biocompatibility assessments were conducted by analyzing human adipose-derived stem cell (hADSC) growth on PLA-CD scaffolds, with results substantiated through cell viability and morphological analyses. hADSCs reached a cell viability of 125% and penetrated throughout the scaffold after 21 days of incubation. These findings underscore the scaffolds’ potential in bone regeneration and fluorescence imaging. The multifunctional nature of the PLA-CD nanocomposite, integrating diagnostic capabilities with tunable properties, positions it as a promising candidate for advancing bone tissue engineering. Our study not only highlights key aspects of the investigation but also underscores the scaffolds’ specific application in bone regeneration, providing a foundation for further research and optimization in this critical biomedical field.

## 1. Introduction

Large bone defects resulting from trauma, congenital anomalies, and diseases such as cancer pose formidable challenges for surgeons. Overcoming these challenges hinges on finding alternatives to conventional treatments, which carry high risks and various limitations, including donor site morbidity, pain, and the potential for rejection. In response to this need, bone tissue engineering (BTE) has emerged as a promising method in recent years, involving the strategic combination of osteogenic progenitor cells, growth factors, and porous biocompatible scaffolds.

For successful three-dimensional (3D) tissue development, biomimetic support is essential, providing a temporary microenvironment for bone cells. An ideal scaffold for bone regeneration should possess key properties, including biocompatibility and porosity to sustain cell proliferation and differentiation, osteoconductivity and osteoinductivity to facilitate the bone healing process, and biodegradability for gradual restoration by the host tissue [1,2,3,4]. Moreover, there is a growing desire for fluorescent scaffolds that can be monitored non-invasively, offering a means to track both bone regeneration and biodegradation processes [5,6].

A myriad of biomaterials has been evaluated for BTE scaffolds, encompassing bioceramic materials, natural polymers, synthetic polymers, and hydrogels. Among these, synthetic polymers, particularly poly(D,L-lactide) acid (PLA), have garnered attention due to their controlled physicochemical and mechanical properties, degradation rate, and ease of processability [7,8,9].

Recent advancements in the field have seen carbon-based nanomaterials (CNMs) gaining considerable interest as candidates for BTE, owing to their excellent biocompatibility, physicochemical properties, remarkable mechanical strength, large specific surface area, and superior optical properties [10,11,12]. Within the realm of CNMs, carbon dots (CDs) have emerged as particularly intriguing, finding applications in various biological contexts due to their high specific surface area, good water solubility, chemical stability, biocompatibility, low toxicity, antimicrobial properties, and biodegradability. CDs exhibit versatile features, including electrical conductivity and stable fluorescence [13].

Notably, the exceptional optical properties and high specific surface area of CDs allow for surface physical and chemical modifications, further enhancing their functionality in biomedical applications [14,15,16,17,18]. A highly efficient protocol has recently been developed to yield decagram-scale quantities of N,S-doped CDs featuring a narrow size distribution and bright emission. These CDs manifest a distinctive nanostructure, exhibiting multicolor emission from blue to near-infrared (NIR) [19,20]. Notably, they have demonstrated the capacity to function as biocompatible self-tracking nanoplatforms for in vivo imaging applications. These unique attributes position CDs as ideal materials for engineering fluorescent scaffolds, demonstrating excellent outcomes in wound healing and bone regeneration. Recent in vitro and in vivo studies have showcased that the incorporation of CDs into composite materials leads to improvements in the degradation rate of PLA scaffolds, mechanical strength, cell adhesion, proliferation, and osteogenic differentiation [21,22,23]. In addition, it has been recently reported that PLA can be engineered using a cost-effective one-pot synthesis methodology for the production of fluorescent highly biodegradable, adhesive, and biocompatible CDs@PLA nanocomposites using the heterophase melt-extrusion transesterification (HMET) process, harnessing CDs as a polar multicolor fluorescent probe filler and PLA as a well-established biocompatible thermoplastic matrix [23]. Heterophase melt-extrusion transesterification has proven capable of promoting CD functionalization with short PLA chains, thus facilitating the integration of PLA-coated CDs uniformly embedded within the PLA matrix.

In this study, we used HMET to obtain a PLA-CD nanocomposite containing 1% multicolor CDs, and we employed this nanocomposite as a starting material for the production of microporous PLA-CD scaffolds with tailor-made properties by using the temperature-induced phase separation (TIPS) technique. Biocompatibility evaluations were carried out through the examination of human adipose-derived stem cell (hADSC) growth on PLA-CD scaffolds, with the outcomes validated through analyses of cell viability and morphology.

## 2. Materials and Methods

### 2.1. Materials

Poly(D,L-lactide) acid terminated (PLA) (Mw 120 kDa), urea (99%), citric acid (99.5%), anhydrous N,N-dimethylformamide (DMF), dichloromethane (DCM), indocyanine green (ICG, 99.5%), Sephadex^®^ G15, Sephadex^®^ G25, dialysis tubing MWCO 2 kDa, phosphate buffer saline (PBS) pH 7.4 (99%), and anhydrous potassium bromide (KBr) (99%) were purchased from Sigma Aldrich (Milan, Italy) and used as received.

Fetal bovine serum (FBS), L-glutamine, penicillin, streptomycin, and amphotericin B were purchased from EuroClone (Milan, Italy). An MTT (3-(4,5-dimethylthiazol-2-yl)-2,5-di- 102 phenyltetrazolium bromide) assay kit was purchased from Merk Life Science S.r.l. (Milan, Italy).

### 2.2. Synthesis of Multicolor Carbon Nanodots (CDs)

CDs of about 5 nm in diameter and with an emission profile within the entire visible region were synthesized via a solvothermal approach employing urea (11.56 g, 0.0577 mol), citric acid (36.95 g, 0.0577 mol), and indocyanine green (100 mg, 0.129 mmol) as precursors in anhydrous DMF (100 mL), as previously detailed (Figure S1a–c) [19]. Briefly, the reaction was maintained at 170 °C for 6 h under controlled pressure conditions, facilitated by the use of a Büchi AG autoclave (Miniclave steel type 3, Gschwaderstrasse, Uster, Switzerland). Following solvent evaporation, the resulting N,S-CDs were obtained as a brownish powder and subsequently purified by size-exclusion chromatography (SEC) utilizing a column packed with Sephadex^®^ G15 and G25. The reaction yield of this process was 43%.

### 2.3. Preparation of the PLA-CD Nanocomposite

The PLA-CD nanocomposite was obtained as previously reported [23]. The main characteristics were extensively studied and are reported in the Supplementary Materials. Briefly, CDs (100 mg, 2.98 mg mL^−1^) were dispersed in acetone/water (95:5) through sonication, and the resulting colloidal dispersion was gradually added to a homogeneous solution of PLA in DCM (20% *w*/*v*, 100 mL), yielding a clear PLA/CD dispersion. The resultant blend was stirred overnight at room temperature. Subsequently, approximately 30 mL of organic solvent was removed using a rotary evaporator, resulting in a slurry that was evenly spread on a glass crystallizer to allow for the gradual removal of solvents at room temperature overnight. The resulting brown PLA/CD film was then cut into small pieces. These PLA/CD pieces (20 g) were subsequently fed into the barrel section of an extruder (Noztek Pro Filament Extruder—London, UK) and extruded at 185 °C, producing filaments of PLA-CD nanocomposite (Figure S2).

### 2.4. Preparation of the Microporous PLA-CD Nanocomposite Scaffolds

The scaffolds were produced using the thermal-induced phase separation (TIPS) technique, maintaining a solvent/non-solvent ratio of 87:13 (1,4-dioxane and water) and using concentrations of PLA-CD of 4%, 4.5%, and 5% *w*/*w*. Twenty-four-well multiwell plates coated with aluminum were employed as molds for the production of 3D microporous scaffolds of about 8 mm in diameter. 1,4-dioxane was preheated to a temperature of 60 °C, and PLA-CD was dissolved under continuous stirring. Then, hot water was added to the PLA solution dropwise, and the dispersion was maintained at 80 °C until a clear solution was obtained. Meanwhile, the 24-well plate was preheated in an oven at 80 °C for approximately 15 min. The homogeneous PLA solution obtained was then added to each well and placed in a −80 °C freezer for about 1 h, reaching the cloudy point after a thermal shock. Subsequently, each scaffold was removed from the mold and immersed in water at room temperature, allowing for the release of the organic solvent and the formation of porous scaffolds. Finally, the scaffolds underwent multiple washes (5 times) with ultrapure water, followed by freeze-drying for 24 h. Yield: 98%.

### 2.5. Evaluation of the CD Encapsulation Efficacy in the Scaffolds

The amount of CDs incorporated during the phase separation process by all PLA-CD scaffolds was calculated spectrophotometrically by dissolving each sample (10 mg) in DCM (5 mL) and recording the absorbance at λ = 450 nm. Measurements were carried out using a single-beam fiber optic spectrophotometer, Star Line ULS2048CLEVO (by Avantes, Apeldoorn, The Netherland), equipped with a dual halogen–deuterium light source covering a spectral range of 200–900 nm. Quartz cuvettes with a 1 cm path length were employed for the spectroscopic measurements. The amount of CDs incorporated in the PLA-CD scaffolds was calculated by comparing the absorption with those of a calibration curve obtained from a pure CD dispersion in DCM (0.01–1 mg mL^−1^, R2 = 0.9974).

### 2.6. Fourier Transform Infrared (FTIR) Spectroscopy Analysis of the PLA-CD Scaffolds

The chemical characterization of all scaffolds was performed using FTIR on a Bruker Alpha II spectrometer (Großweikersdorf, Austria) using a transmission geometry. Samples were obtained as KBr pellets with a concentration of 0.2% *w*/*w*, and measurements were performed at room temperature, with the spectra being recorded within the range 400–4000 cm^−1^ (scan time of 24 and resolution of 4 cm^−1^).

### 2.7. Differential Scanning Calorimetry (DSC) and Thermal Gravimetric Analysis (TGA)

DSC/TGA analyses of all scaffolds were carried out using a DSC/TGA 131 EVO (by Setaram Instruments, Caluire, France). Briefly, either PLA-CD 4%, PLA-CD 4.5%, or PLA-CD 5% (~5 mg) scaffolds were placed in an aluminum crucible under a nitrogen atmosphere (flow 10 mL min^−1^), and then a heating rate of 10 °C min^−1^ in the range of 30–300 °C was applied. For comparative purposes, all thermograms were normalized to a unit weight.

### 2.8. Micro-Computed Tomography Analysis (Micro-CT)

The internal microporous organization and potential structural defects in PLA-CD 5% scaffolds were investigated through Micro-CT acquisitions, utilizing a Bruker Skyscan 1272 system (Großweikersdorf, Austria). The measurements were conducted at a voltage of 40 kV and a current of 250 µA, with a rotation step of 0.2° and a resolution of 4.5 µm.

### 2.9. Scanning Electron Microscopy (SEM)

Scanning electron microscopy measurements were performed on the PLA-CD 4%, PLA-CD 4.5%, and PLA-CD 5% scaffolds, named 4%, 4.5%, and 5%, using a Phenom Desktop Scanning Electron Microscope operating at 15 kV (Phenom, Berlin, Germany). All samples were dried before the measurements, deposited on carbon tape, and observed without previous treatments.

### 2.10. Degradation Kinetics under Physiological Conditions

The degradation kinetics were established for the PLA-CD 4%, PLA-CD 4.5%, and PLA-CD 5% scaffolds under physiological conditions. In particular, each scaffold was dried, weighed, and incubated with PBS pH 7.4 (40 mL) in an orbital shaker at 37 °C for up to 180 days. At predefined intervals, samples were retrieved, gently wiped with a paper filter, and re-weighed. Following each measurement, the samples were returned to the test tube and maintained at 37 °C before subsequent assessments. The percentage of *Sample weight* was calculated as the mean value of six measures using Equation (1):

(1) $$Sample\;weight\;\left(\%\right)=\frac{W_{f}}{W_{i}}\times 100$$

where $W_{f}$ is the residual weight obtained at the measurement time and $W_{i}$ is the initial weight.

### 2.11. Rheological Studies

A viscoelastic analysis was performed to investigate the behavior of the 5% *w*/*w* PLA-CD scaffold, and the results were compared with those of the scaffold composed solely of PLA, as the temperature varied. This assessment was conducted using a Discovery HR-2 rotational rheometer (by TA Instruments, New Castle, DE, USA). All measurements were performed using a parallel plate geometry (d = 20 mm) with a gap of 250 µm and a temperature range from 15 °C to 180 °C. The linear viscoelastic region was established by applying a shear strain ramp from 0.01 to 10% at a frequency of 1 Hz. The compressive force applied during the measurements was 0.0 N. A deformation of 0.1% was selected for frequency sweep analysis across the frequency range from 0.1 to 10 rad s^−1^. Each measurement was performed three times on different samples.

### 2.12. Optical Characterization of the PLA-CD 5% Scaffold

The optical absorption measurements were performed in a 1 cm quartz cuvette using a Star Line ULS2048CLEVO single-beam optical fiber spectrophotometer (Avantes) equipped with a dual halogen–deuterium light source in the spectral range of 200–900 nm. Steady-state emission spectra were recorded on an intensified CCD camera integrating the signal during temporal windows of 1 ms from the excitation laser pulse. A tunable laser (OPO—optical parametric oscillator) providing 5 ns pulses at 10 Hz has been employed to excite the samples.

Quantum yield (QY) values were estimated by measuring the emission and absorption of the sample in an integrating sphere. The samples were excited by laser diodes peaking at 405 nm and at 532 nm.

### 2.13. Fluorescence Microscopy

The emission behavior of the PLA-CD 5% scaffold was established by fluorescence microscopy using an AxioCam MRm (by Zeiss, Jena, Germany). Given the presence of fluorescent multicolor CDs incorporated into the PLA matrix, micrographs were acquired from a small surface and lateral portion of the 5% *w*/*w* PLA-CD scaffold using the DAPI, FITC, and TxR channels.

### 2.14. Biological Characterization

Human adipose-derived mesenchymal stem cells (hADSCs) were obtained from adipose tissue biopsies as previously reported [24]. Briefly, adipose tissue biopsies were mechanically dissociated into smaller pieces and enzymatically digested at 37 °C with a collagenase I solution (Gibco, Thermo Fisher Scientific, Waltham, MA, USA) for two hours. Then digested fragments were centrifuged, the floating fat was removed, and the supernatant was filtered and newly centrifuged. Finally, the pellet containing the cells was resuspended in ADSC basal medium (Lonza Group Ltd., Basel, Switzerland) supplemented with 2.5 mM L-glutamine (Merk Life Science S.r.l., Milan, Italy), 10% fetal bovine serum (FBS, Merk Life Science S.r.l., Milan, Italy), and 1% penicillin/streptomycin/amphotericin (Merk Life Science S.r.l., Milan, Italy) and incubated at 37 °C in a humidified atmosphere containing 5% CO_2_. The next day, the medium was replaced to eliminate nonadherent cells, and hADSCs were maintained until 80–90% confluence. The medium was replaced two or three times a week.

#### 2.14.1. Cell Viability Assay on PLA-CD 5% Scaffolds

A cell viability assay of hADSCs seeded on PLA-CD 5% scaffolds was performed using an MTT (3-(4,5-dimethylthiazol-2-yl)-2,5-diphenyltetrazolium bromide) assay kit (Merk Life Science S.r.l., Milan, Italy). Specifically, 5 × 105 cells resuspended in 50 μL of medium were slowly drip-seeded onto the PLA/CD scaffolds, in 24-well culture plates, and incubated in a humidified atmosphere containing 5% CO_2_ at 37 °C for 4 h. Four hours after, ADSC basal medium was added to wholly cover the scaffolds, and the plates were re-incubated, as above, for 1, 7, 14, and 21 days. The fresh medium was replaced two or three times a week. At the end of each timepoint, ADSC basal medium was removed from each well, replaced with MTT solution (1 mg/mL in FBS-free medium), and incubated at 37 °C and 5% CO_2_ for 2 h. Then, the MTT solution was eliminated, each well was washed 2 times with PBS 1×, and the produced formazan crystals were dissolved using DMSO. Finally, the absorbance at 540 nm was read using a synergy HT plate reader (BioTek Instruments, Inc., Tigan Street Winooski, VT, USA). For each analyzed timepoint, biological tests were performed in triplicate, and cell viability differences were assessed using the one-way ANOVA test with the Holm test as a post hoc test for multiple comparisons.

#### 2.14.2. Histological Analyses on PLA-CD 5% Scaffolds and hADSCs

At the end of 1, 7, 14, and 21 days, scaffolds seeded with hADSCs were fixed in 4% PFA, dehydrated, embedded in paraffin, cut into 8μm-thick transversal sections, and mounted on slides. Afterward, the sections were dewaxed in xylene, hydrated with graded ethanol, and stained with H&E to evaluate cell morphology and the presence or absence of morphological alterations. The slides were analyzed by using a Leica DMI 4000B microscope (Leica Microsystems S.r.l., Milano, Italy). At least five sections/scaffold were analyzed.

#### 2.14.3. Statistical Data Analysis

Data were analyzed either as raw data or as mean ± standard error (SE), as appropriate. Differences between several time points of hADSC-seeded PLA/CD scaffolds were evaluated by using one-way ANOVA with post hoc Holm test, where appropriate. *p* < 0.05 was considered significant.

## 3. Results and Discussion

N,S-co-doped carbon nanodots of 5.3 ± 0.4 nm in diameter exhibiting tunable multicolor emission (CDs) were synthesized via the solvothermal decomposition of urea, citric acid, and indocyanine green (ICG) in DMF, as reported in our previous work (Figure S1a–c) [19,23]. In a nutshell, a nitrogen–sulfur-co-doped carbon crystalline nanocore was obtained using equimolar amounts of citric acid and urea, with a non-stoichiometric quantity of ICG serving as the sulfur donor. This methodology not only enhanced the optical properties of CDs in terms of multicolor fluorescence (ranging from blue-QY 12% to NIR-QY 1%) but also resulted in CDs with distinct surface polar groups exploitable for subsequent surface functionalization, including carboxyl, hydroxyl, amide, and sulfonate/sulfoxide (Figure S1c) [19]. In particular, hydroxyl groups present on the surface of these peculiar CDs have been successfully used for initiating one-pot solid/liquid heterophase transesterification of polyesters, such as PLA, through melt-extrusion processes (Figure S2). We pioneered an inventive one-pot synthesis for creating fluorescent biodegradable CD-PLA nanocomposites through a scalable heterophase melt-extrusion transesterification process, providing exceptional thermal stability allowing additive manufacturing [19]. The resultant PLA-CD nanocomposite demonstrates inherent self-tracking capabilities with stable multicolor fluorescence in the solid state, accelerates PLA degradation, and promotes enhanced cell adhesion, positioning it as a compelling choice for the advanced production of biomedical devices through 3D printing applications.

Here, we advanced the development of this innovative PLA-CD nanocomposite to produce microporous scaffolds using thermally induced phase separation (TIPS). These scaffolds feature multicolor fluorescence (i.e., self-tracking fluorescence), tailored porosity, tunable biodegradation, excellent mechanical performance, and the ability to promote osteoblast adhesion and proliferation.

### 3.1. Preparation of Fluorescent Scaffolds with Controlled and Interconnected Porosity

**Figure 1:**
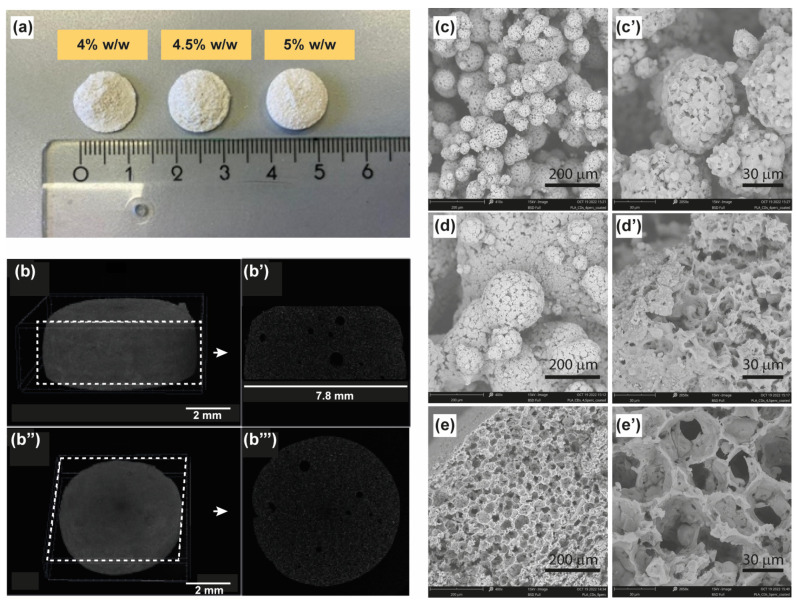
(**a**) Illustration of typical PLA-CD scaffolds obtained using TIPS starting from a dispersion of PLA-CD nanocomposite in DCM at increasing concentration on a weight basis. (**b**–**b′′′**) Micro-CT micrographs obtained for the PLA-CD 5% scaffold. (**c**,**c′**) Scanning electron microscopy (SEM) micrographs of the PLA-CD 4% scaffold, (**d**,**d′**) PLA-CD 4.5% scaffold, and (**e**,**e′**) PLA-CD 5% scaffold.

We harnessed the principle of the heterophase melt-extrusion transesterification process to promote solvent-free direct transesterification between the hydroxyl groups on the surface of carbon dots (CDs) and the ester bonds within PLA chains. This method resulted in the formation of CD-PLA conjugates seamlessly integrated into the hydrophobic matrix of unbonded PLA chains (Figure S2) [23]. The outcome is a remarkably uniform nanocomposite material exhibiting thermostable bright multicolor fluorescence which can be used in thermal manufacturing processes such as 3D printing and TIPS. Among these, TIPS was selected as a simple and scalable method to produce microporous CD-PLA-based scaffolds by varying the concentration of the starting nanocomposite from 4% to 5%. Specifically, we chose three concentrations (i.e., 4%, 4.5%, and 5%) based on prior studies that consistently demonstrated improved porosity and mechanical properties within this range. This selection was made while utilizing a combination of 1,4-dioxane and water 87:13 *v*/*v* as the solvent and non-solvent, respectively, in accordance with established practices [25,26]. The fabrication of the scaffolds involved the use of dedicated 24-well plates preheated in an oven to 70 °C for approximately 15 min and lined with aluminum, with each well capable of holding 2 mL of the starting nanocomposite dispersion in 1,4-dioxane/water at 60 °C. The hot ternary solution of the nanocomposite was then rapidly frozen in a −80°C freezer for about 1 h. The drastic temperature decrease resulted in the formation of discoid porous scaffolds at the bottom of the plate, which was washed with water several times and freeze-dried to remove the residual solvents (Figure 1a). These observed changes can be attributed to the precipitation of the nanocomposite and the concurrent crystallization of the solvent (1,4-dioxane) triggered by the drastic temperature shift. As a consequence, the porosity and robustness of the scaffolds depend on the initial concentration of the nanocomposite.

### 3.2. Physicochemical Characterization of the PLA-CD Scaffolds

#### 3.2.1. Morphological Characterization

The induced thermal shock to the nanocomposite dispersion caused a structural change in the polymeric colloidal dispersion as well as the crystallization of the binary solvent mixture, with obvious structural implications in the 3D scaffolds prepared. On a macroscopic scale, scaffolds produced with the highest concentration tested (5%) exhibited a porous, rigid sponge-like structure characterized by a well-established morphology. In contrast, scaffolds derived from the 4% and 4.5% nanocomposite dispersions appeared exfoliated and displayed irregularities (Figure 1a). The macroscopic architecture of the PLA-CD 5% scaffold was then studied via micro-computed tomography analysis (Micro-CT), as reported in Figure 1b–b′′′, where a compact and porous macrostructure, with a rough surface (Figure 1b,b′′) and without fractures, can be easily distinguished. From the analysis of longitudinal and transversal sections (Figure 1b’,b′′′) one can observe the presence of some occasional bubbles of about 50–100 μm and extensive micrometric pores beyond the resolution of the technique adopted. It might be deduced that the drastic temperature decrease of the ternary system led to a transformation from an irregular distribution to a reticulated and oriented microstructure, resulting in the formation of the porous scaffold at the bottom of the mold. These observed changes can be attributed to the precipitation of the nanocomposite and the concurrent crystallization of the solvent (1,4-dioxane) triggered by the drastic temperature shift. As a consequence, the porosity and robustness of the scaffolds depend on the initial concentration of the nanocomposite.

This hypothesis was further assessed by analyzing the microstructure of the scaffolds using electronic scanning microscopy (SEM). The PLA-CD 4% scaffold is clearly formed by clustered porous microparticles of about 50 μm with a broad size distribution, without a distinguishable hierarchically organized microstructure (Figure 1c,c′). SEM micrographs of the PLA-CD 4.5% scaffold display that the adopted conditions for the preparation of this scaffold also imply the formation of fused microparticles without a distinct spatial organization (Figure 1d,d′), but with qualifications. In particular, the PLA-CD 4.5% scaffold consists of heterogeneous microparticles of 200 μm in diameter, showcasing regularly arranged pores of about 1–2 μm. It is noteworthy that both the PLA-CD 4% and PLA-CD 4.5% scaffolds exhibit a discontinuous bulk structure, contributing to the observed high macroscopic fragility. On the contrary, the PLA-CD 5% scaffold presents a continuous bulk structure with interconnected pores of about 40 μm (Figure 1e,e′). The well-organized microstructure observed explains the robust macroscopic features of the PLA-CD 5% scaffold. Moreover, the existence of regularly arranged pores with an appropriate diameter is crucial to facilitate extensive cell migration during plasticity events inherent in bone regeneration. Indeed, pores play a pivotal role in the process of bone regeneration, enabling the exchange of nutrients and oxygen and removal of waste products and facilitating the ingrowth of bone tissue and vasculature within the scaffold [27].

#### 3.2.2. Chemical Characterization

**Figure 2:**
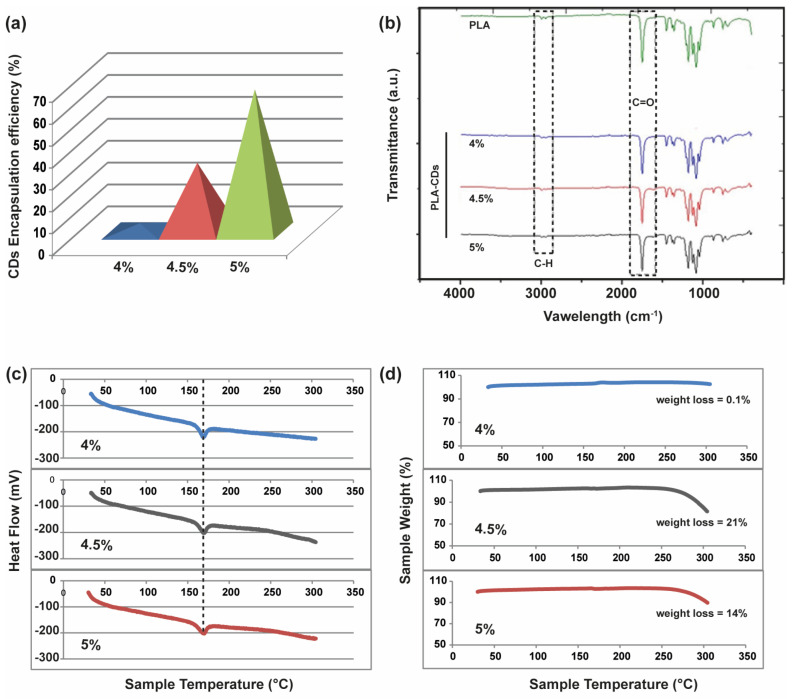
Chemical characterization of the PLA-CD scaffolds obtained using TIPS from the 4%, 4.5%, or 5% dispersion in DCM: (**a**) determination of the percentage of encapsulated CDs with respect to the theoretical; (**b**) FTIR compared with the virgin PLA scaffold; (**c**) thermograms obtained using differential scanning calorimetry (DSC) (endothermic transitions on the bottom; heating rate = 10 °C min^−1^); (**d**) thermal stability profile obtained using thermal gravimetric analysis (heating rate = 10 °C min^−1^).

With the aim of understanding the effective incorporation of CDs in the microporous PLA-CD scaffolds, the amount of CDs was established by dissolving each scaffold in DCM and measuring the absorbance at 450 nm. Thus, the amount of CDs in the nanocomposite scaffolds was calculated by comparing the absorbance with that of a calibration curve of plain CDs (Figure 2a). Surprisingly, the amount of CDs incorporated in the scaffolds, after the TIPS process, increased with the concentration of the starting PLA-CD solution. The encapsulation efficiency passed from 3.8% to 68% for the PLA-CD 4% and PLA-CD 5%, respectively. This can be ascribed to the migration and extraction of CDs from the nanocomposite during the fast phase separation that occurred at low temperatures. This phenomenon is primarily attributed to the swift diffusion of ultrasmall nanoparticles, a characteristic notably enhanced by the utilization of a diluted polymer solution with lower viscosity (i.e., PLA-CD 4%).

The formation of homogeneous PLA-CD nanocomposite scaffolds was assessed via FTIR analysis. As shown in Figure 2b, the PLA-CD scaffolds exhibit the characteristic vibrations associated with pure PLA, which is the predominant component of the scaffolds in terms of weight. In particular, there are the vibration bands relative to carbonyl functions (1746 cm^−1^) and methylene protons (2984 cm^−1^) of PLA. Furthermore, the PLA-CD 5% peaks within the fingerprint region (1129 and 1083 cm^−1^) exhibit a distinct asymmetry compared to the PLA spectrum, indicating an increase in the OH bending due to transesterification occurring during the extrusion process [23]. Overall, the FTIR spectra show homogeneous samples prevailingly constituted by the bare PLA matrix.

To assess the thermal stability of the PLA-CD scaffolds and the effect of different amounts of filler, namely CDs, in the nanocomposite matrix, DSC/TGA analysis was conducted over a temperature range of 25–300 °C with a heating rate of 10 °C min^−1^. As shown in Figure 2c, there is a typical endothermic peak at 160 °C in all DSC thermograms, attributable to the melting point (T_m_) of the PLA matrix. According to the FTIR study, all samples seem characterized by the predominant behavior of the bare PLA chains. However, a very different behavior is observed in the TGA curves for the three samples (Figure 2d). Specifically, no significant weight loss is registered for the PLA-CD 4% sample up to 300 °C, while a weight loss of about 21% and 14% can be observed from 270 to 300 °C for the PLA-CD 4.5% and PLA-CD 5% samples, respectively. This remarkably different behavior can be due to the decomposition of PLA chains induced by the presence of a higher amount of CDs in the PLA-CD 4.5% and PLA-CD 5% samples (Figure 2a). The surface polar groups of CDs are quite labile at temperatures higher than 200 °C, thus impinging on the PLA-CD nanocomposite stability [28].

### 3.3. Optical Characterization of the PLA-CD Scaffolds

**Figure 3:**
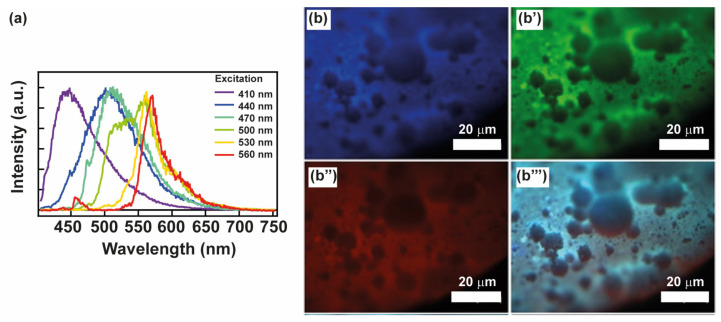
(**a**) Fluorescence emission spectra of the PLA-CD 5% scaffold under excitation at 410, 440, 470, 500, 530, and 560 nm. (**b**–**b′′′**) Fluorescence microscopy of the PLA-CD 5%: micrographs captured exploiting the emission behavior of CDs entrapped in the PLA matrix, and using (**b**) blue, (**b′**) green, and (**b′′**) red emission channels; (**b′′′**) merge of all fluorescence channels.

Considering that crystalline CDs exhibit a higher percentage of sp^2^ domains compared to amorphous CDs, they are more susceptible to self-quenching phenomena induced by surface interactions at short distances in the solid state [29]. Nevertheless, surface passivation with PLA chains guarantees an excellent emission profile for CDs, even in the solid state [23]. This is due to the presence of a polymer shell at the CDs’ surface and their consequent exceptional dispersion within the PLA solid matrix (Figure S2), significantly mitigating π-π core interactions or aggregation phenomena [30]. In principle, this feature should be maintained after the TIPS process, even if a slight migration of CDs towards the binary solvent mixture and throughout the polymer matrix occurs (Figure 2a). The emission fluorescence spectra of the selected PLA-CD 5% scaffold obtained at increasing excitation wavelengths (λex, 410–560 nm) are reported in Figure 3a. The spectrum still displays the typical tunability of multicolor CDs owing to many electronic transitions in the entire visible range. Thus, the fundamental optical properties of CDs, that is the fluorescence tenability and bright emission, are preserved in the composite PLA-CD 5% scaffold, implying that TIPS is a good technique for producing highly emissive microporous scaffolds using the PLA-CD nanocomposite. To evaluate if the PLA-CD 5% scaffold is a good candidate for image-guided bone tissue engineering applications, we also performed fluorescence microscopy measurements using the blue, green, and red fluorescence channels (Figure 3b–b′′′).

Remarkably, the PLA-CD 5% scaffold is observable in all channels owing to the inherent self-fluorescence of the encapsulated CDs. This characteristic provides the opportunity to discern various structural details in a non-invasive way (i.e., without sample preparation and manipulation, atmospheric pressure and temperature). In detail, the microporous and rough nature of the surface is quite visible using both the blue, green, and red emission and the macroscopic features. Thus, fluorescence imaging can be employed as a powerful and discrete technique to monitor the scaffold allocation and microstructure upon implantation.

### 3.4. Degradation Kinetics under Physiological Conditions

**Figure 4:**
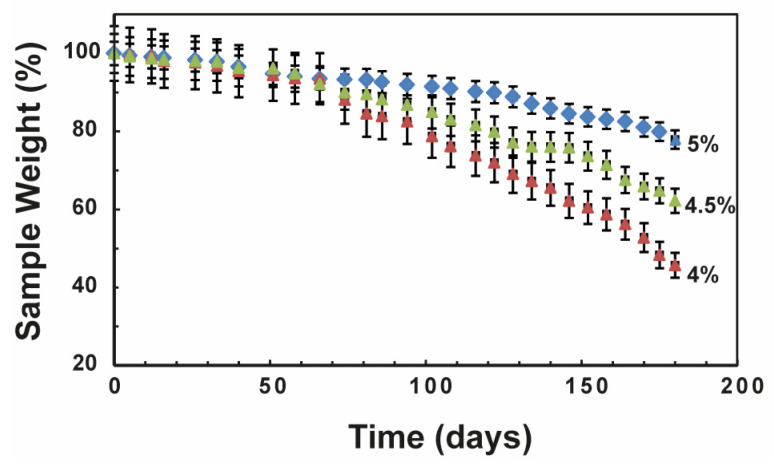
Degradation kinetics of the PLA-CD scaffolds obtained using TIPS from the 4%, 4.5%, or 5% dispersion in DCM: the residual sample weight is depicted as a function of the incubation time at 37 °C in PBS pH 7.4.

Typically, PLA is considered to be liable to bulk degradation under physiological conditions. Nonetheless, PLA demonstrates substantial resistance to hydrolysis, resulting in the persistence of PLA implants (such as orthopedic screws) for extended periods in vivo [31,32]. There is a growing demand for PLA nanocomposites with adjustable stiffness and degradation rates to meet various requirements in bone regeneration applications. Hence, we investigated the degradation rate of all PLA-CD scaffolds in PBS pH 7.4 at 37 °C over a period of 6 months. In particular, we measured the sample weight percentage of all scaffolds incubated under physiological conditions over time (Figure 4). As expected, all samples underwent a slow degradation process with a porosity-dependent slope. The PLA-CD sample with higher porosity, namely PLA-CD 4%, rapidly degraded during 6 months of incubation, displaying a weight loss of 58%. The weight loss registered for the PLA-CD 4.5% sample, endowed with middle porosity, is much more moderate (about 37%). However, the sharp slope of the degradation curves for these samples suggests the loss of the main internal architecture and the formation of collapsed structures. Meanwhile, lower weight loss is clearly shown by the scaffold with interconnected pores and lower average porosity (PLA-CD 5%). The latter sample maintains a controlled degradation rate for up to 6 months of incubation, which implies a stable structure during the degradation processes. The maintenance of a rigid architecture was also confirmed by fluorescence microscopy after 6 months of incubation in PBS at 37 °C (Figure S3). The bright multicolor fluorescence was preserved after 6 months and allowed the tracking of morphological variations of the original scaffold. The controlled degradation rate under physiological conditions is required for in vivo applications since the initial properties should be kept during bone regeneration [33,34].

### 3.5. Viscoelastic Characterization of the PLA-CD 5% Scaffold

**Figure 5:**
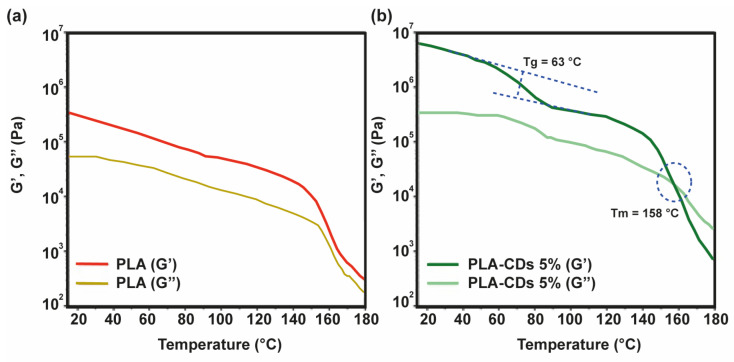
Rheological analysis performed for the bare PLA (**a**) and the PLA-CD 5% scaffolds (**b**) as a function of the sample temperature. The elastic (G′) and dissipative (G′′) moduli were obtained in the linear viscoelastic region.

The rheological characteristics of polymeric scaffolds play a crucial role in establishing the structure and, consequently, the potential applications of polymeric scaffolds. To delineate the linear viscoelastic zone, the samples were subjected to a strain sweep analysis, wherein they were subjected to various shear strains at a frequency of 1.0 Hz, while a compressive force of 0.0 N was applied. The shear storage modulus (G′) remained relatively constant below 1% shear strain. Beyond 1% shear strain, G′ exhibited a decrease with increasing shear strain, indicating a transition from linear to non-linear behavior [35]. Consequently, a deformation of 0.1% was selected for subsequent tests to ensure that each measurement was conducted within the linear viscoelastic region. Each sample underwent a frequency sweep across the frequency range from 0.1 to 10 rad s^−1^. As depicted in Figure 5a, the storage modulus (G′) of the bare PLA scaffold was about 5 × 10^5^ Pa at 25 °C. This value decreased to 6 × 10^4^ Pa at 150 °C and underwent a sharp decrease at 160 °C. The well-developed polymer structure was also maintained during the melting transition (155–180 °C) of the sample, since G′ was always higher than G′′. The rheological behavior of the PLA-CD 5% scaffold displays some characteristic features (Figure 5b).

Specifically, the initial storage modulus (G′) was about two orders of magnitude higher, 8 × 10^7^ Pa, compared to the bare PLA scaffold at the same temperature (25 °C). This means that the mechanical performance of the nanocomposite scaffold is higher than that of virgin PLA. This can be due to the formation of a mechanically robust network between the CD shell and the PLA matrix. Moreover, it can be distinguished that there was a sigmoidal decrease in the elastic modulus at about 63 °C, attributable to the glass transition (T_g_), and a cross-over between the storage modulus (G′) and the loss modulus (G′′) at 158 °C (G′ < G′′) ascribable to the melting processes taking place within the range 155–180 °C. This implies that the PLA-CD nanocomposite behaves as a highly viscous fluid in the melting state, instead of a gel-like structure, owing to the diffusion of the PLA-functionalized CD filler and a fast generation of low-molecular-weight PLA chains due to thermo-oxidative degradation (Figure S2) under atmospheric conditions [36].

### 3.6. Biological Characterization of the PLA-CD 5% Scaffold

**Figure 6:**
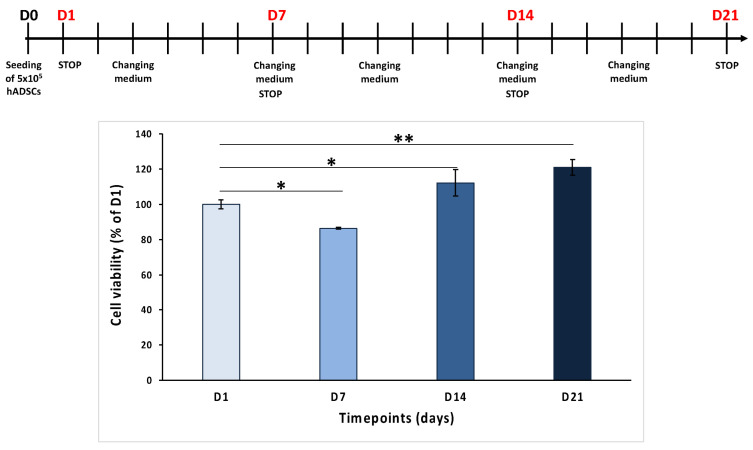
MTT assay of hADSCs grown on PLA-CD 5% scaffolds for 1, 7, 14, and 21 days. ANOVA test *p* values are reported, and * *p* < 0.05 or ** *p* < 0.01 indicates significant differences between scaffolds as reported by the Holm post hoc test.

To assess the biocompatibility of the selected PLA-CD 5% scaffolds, human adipose-derived stem cells (hADSCs) were cultured on the scaffolds for many days (1, 7, 14, and 24 days), and a cell viability assay was conducted. The results reported in Figure 6 show that the cell number increases over time from day 1 (D1) to day 21 (D21), except for day 7 (D7), when a reduction in cell viability was noted, probably due to a different adhesion of the cells on these scaffolds. In detail, the MTT values were 86.4 ± 0.6% on D7, 112.1 ± 7.5% on D14, and 121 ± 4.5% on D21, compared to 100 ± 2.6% on D1. These results suggest that PLA-CD 5% scaffolds are cytocompatible and that hADSCs seeded on them proliferate over time.

The data obtained from MTT analysis showed the good biocompatibility of the tested PLA-CD 5% scaffold, in agreement with the ISO Standard 10993-5 of 2009 establishing that only a cell viability reduction of more than 30% is considered cytotoxic [37].

**Figure 7:**
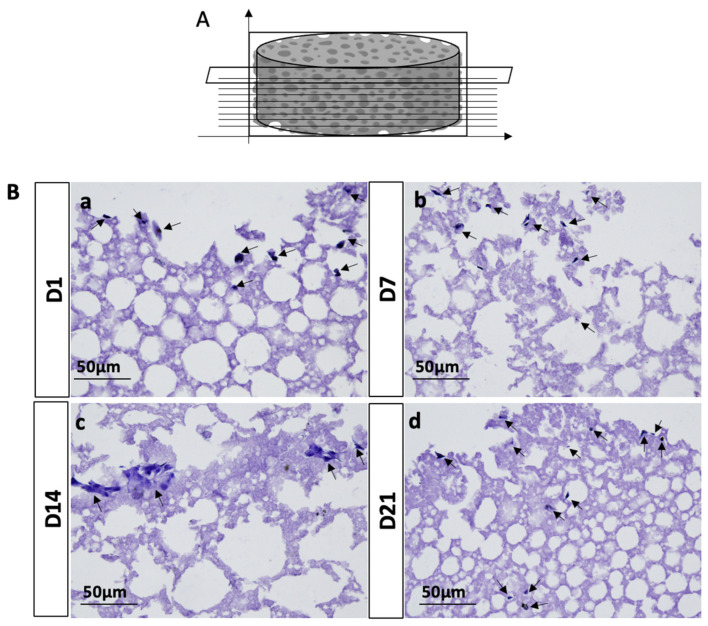
Morphological characterization of hADSC-seeded PLA-CD 5% scaffolds after 1 (**a**), 7 (**b**), 14 (**c**), and 21 (**d**) days of cell culture. (**A**) Schematic drawing of hADSC-seeded PLA-CD 5% scaffolds, showing the transverse cutting planes. (**B**) Representative images of hADSC-seeded PLA-CD 5% cross-sections stained with H&E. Black arrows: hADSCs. Scale bar: 50 µm.

To further evaluate the ability of seeded hADSCs on PLA-CD 5% scaffolds to penetrate and propagate into the whole biomaterial structure, we performed H&E staining. The results obtained from the morphological characterization of hADSC-seeded PLA-CD 5% scaffolds after 21 days of cell culture displayed that cells were present in all the analyzed transversal sections, suggesting that the seeded cells have a good ability to penetrate and invade the whole scaffold structure (Figure 7a–d).

**Figure 8:**
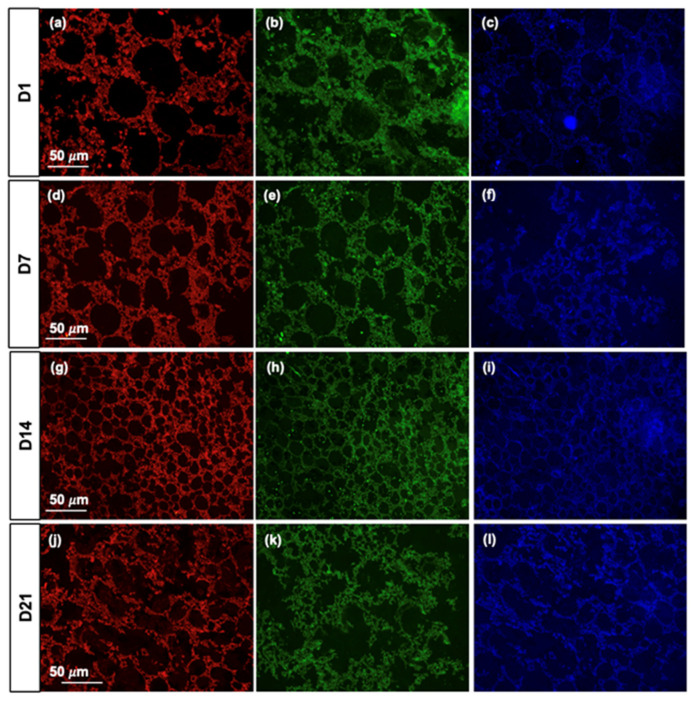
Fluorescence micrographs of PLA-CD 5% slides after 1, 7, 14, and 21 days of cell culture. Red-emitting PLA-CD 5% (**a**,**d**,**g**,**j**), green-emitting PLA-CD 5% (**b**,**e**,**h**,**k**), and blue-emitting PLA-CD 5% (**c**,**f**,**i**,**l**). Scale bar: 50 µm.

In addition, we evaluated the fluorescence properties of PLA-CD 5% scaffolds upon incubation with cells under physiological conditions for up to 2 days. As can be seen in Figure 8a–l, PLA-CD 5% scaffolds preserve the fluorescence property of CDs at every time point analyzed, suggesting their stability under physiological conditions and relevance in non-invasive tissue engineering monitoring applications. The data derived from both cell viability and morphological characterization analyses align with prior studies, indicating that PLA scaffolds exhibit the capability to support cell adhesion, growth, and proliferation [38]. Moreover, the incorporation of highly stable fluorescent CDs into a PLA polymer matrix offers a long-term non-invasive cell–scaffold interaction helpful in analyzing tissue regeneration kinetics [34].

## 4. Conclusions

In conclusion, this work shows the possibility of designing promising biodegradable scaffolds with both bioactive and bioimaging properties useful for monitoring the healing process and promoting bone regeneration. The developed PLA-CD nanocomposite scaffolds, particularly the PLA-CD 5% variant, demonstrated promising attributes for biomedical applications. The macroscopic observations revealed distinct structural differences among various concentrations, with the 5% scaffold exhibiting a continuous bulk structure and interconnected pores. These structural characteristics are critical, as they directly impact cell migration and tissue ingrowth, crucial elements in bone regeneration.

The comprehensive characterization, encompassing DSC/TGA analyses, rheology, FTIR studies, and degradation assessments in physiological conditions, provided valuable insights into the material’s mechanical behavior, microstructure, and stability over time. Notably, the PLA-CD 5% scaffold showcased excellent self-fluorescence, enabling detailed visualization in various channels and offering a means to discern structural intricacies.

Furthermore, the biocompatibility evaluation involving hADSC growth on PLA-CD 5% scaffolds, supported by cell viability and morphological analyses, underscored the scaffold’s ability to sustain cell adhesion, growth, and proliferation. This aligns with previous findings on the favorable biocompatibility of PLA scaffolds.

Overall, the multifunctional characteristics of the PLA-CD nanocomposite, combining diagnostic capabilities with tunable properties, make it a promising candidate for applications in tissue engineering and regenerative medicine. Further studies can delve into optimizing specific parameters for enhanced performance and explore the potential therapeutic applications of this innovative nanocomposite scaffold.

## Data Availability

Data are contained within the article and Supplementary Materials.

## Supplementary Materials

The following supporting information can be downloaded at https://www.mdpi.com/article/10.3390/ma17020449/s1. Figure S1: Scheme of synthesis, emission profile, and size distribution of CDs; Figure S2: Heterophase melt-extrusion transesterification (HMET) process adopted for the synthesis of the PLA-CD nanocomposite. Figure S3. Fluorescence microscopy of the PLA-CDs 5% scaffold after 6 months of degradation in PBS pH 7.4 at 37 °C.
