# Supplementary material for: Microporous Fluorescent Poly(D,L-lactide) Acid–Carbon Nanodot Scaffolds for Bone Tissue Engineering Applications
Source: Materials (Basel). 2024 Jan 17;17(2):449. doi: 10.3390/ma17020449 (PMC10820564; doi:10.3390/ma17020449)
Supplement: Supplementary file 1 [file materials-17-00449-s001.zip › materials-2815198-supplementary.pdf]

Supplementary Materials

Microporous Fluorescent Poly(D,L-lactide) acid-Carbon Nanodots Scaffolds for Bone Tissue Engineering Applications

Nicolò Mauro [1]*, Giovanna Calabrese [2], Alice Sciortino [3], Maria G. Rizzo [2], Fabrizio Messina [3], Gaetano Giammona [1] and Gennara Cavallaro[1]

[1] Department of "Scienze e Tecnologie Biologiche Chimiche e Farmaceutiche" (STEBICEF), Università degli Studi di Palermo, Via Archirafi 32, Palermo, 90123, Italy; gaetano.giammona@unipa.it, nicolo.mauro@unipa.it, gennara.cavallaro@unipa.it, Department of Chemical, Biological, Pharmaceutical and Environmental Sciences, University of Messina, Viale Ferdinando Stagno d'Alcontres, 31, 98168 Messina, Italy
[2] Department of Chemical, Biological, Pharmaceutical and Environmental Sciences, University of Messina, Viale Ferdinando Stagno d'Alcontres, 31, 98168 Messina, Italy
[3] Department of Chimica e Fisica "E. Segrè", Università degli Studi di Palermo, Via Archirafi 36, Palermo, 90123, Italy; alice.sciortino02@unipa.it, fabrizio.messina@unipa.it
* Correspondence: nicolo.mauro@unipa.it; Tel.: +39-09123891908

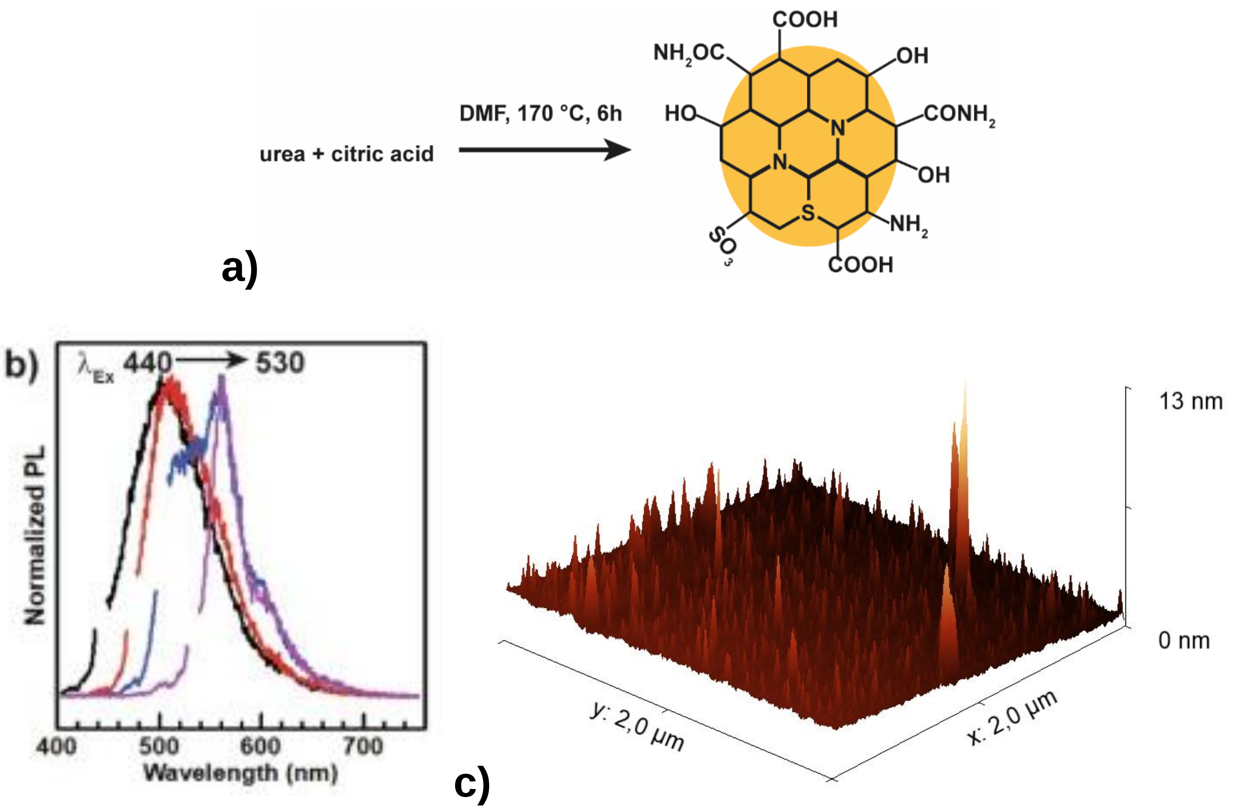

Figure S1. Scheme adopted for the synthesys of S,N-doped CDs (CDs) (**a**). Atomic force microscopy of the CDs used; average diameter of 5.3 ± 0.4 nm (**b**). Emission profile of the CDs used (**c**)

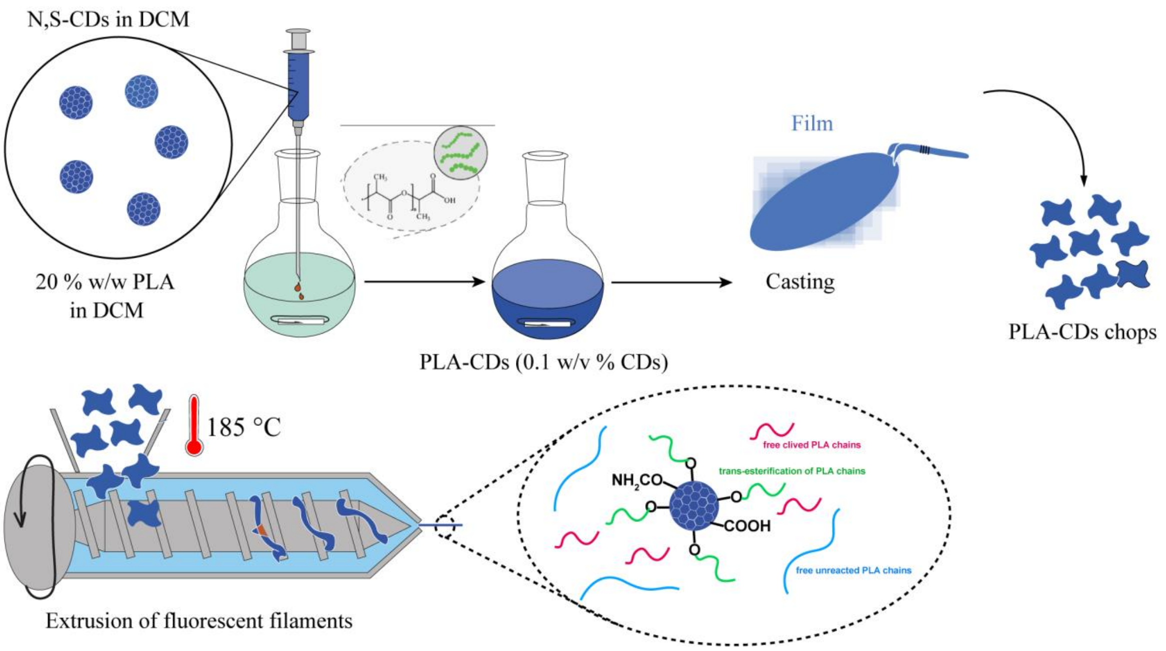

Figure S2. Schematic representation of the melt-extrusion transesterification process adopted for the preparation of the PLA-CDs nanocomposite

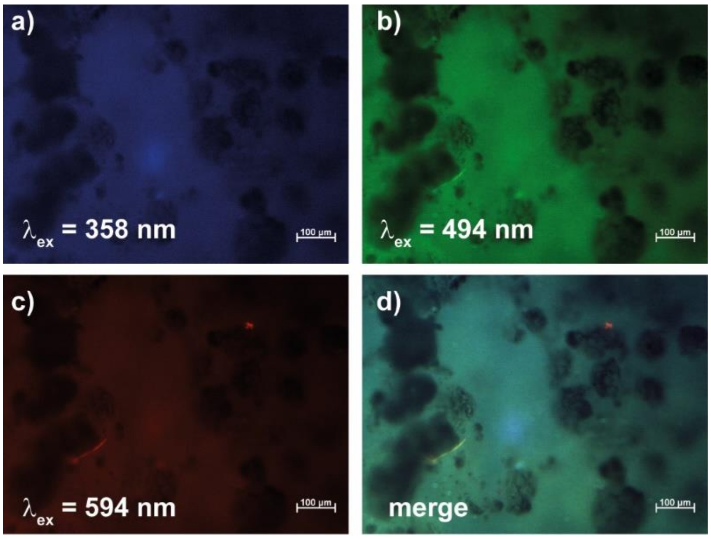

Figure S3. Fluorescence microscopy of the PLA-CDs 5% scaffold after 6 months of degradation in PBS pH 7.4 at 37 °C: a) DAPI channel, b) FITC channel, c) TxR channel, d) merge of all channels.
